# CES1 is associated with cisplatin resistance and poor prognosis of head and neck squamous cell carcinoma

**DOI:** 10.32604/or.2024.052244

**Published:** 2024-11-13

**Authors:** CHUAN JIANG, CHUNLEI LIU, XI YAO, JINGYA SU, WEI LU, ZHENGBO WEI, YING XIE

**Affiliations:** 1 Key Laboratory of Early Prevention and Treatment for Regional High Frequency Tumor (Guangxi Medical University), Ministry ofEducation, Nanning, 530000, China; 2 Life Sciences Institute, Guangxi Medical University, Nanning, 530000, China; 3 Department of Head and Neck Tumor Surgery, Affiliated Tumor Hospital of Guangxi Medical University, Nanning, 530000, China

**Keywords:** Carboxylesterase 1 (CES1), Head and neck squamous cell carcinoma (HNSCC), Chemoresistance, Cisplatin, Smoking, Prognosis

## Abstract

**Background:**

Head and neck squamous cell carcinoma (HNSCC) is a prevalent form of cancer globally, with chemoresistance posing a major challenge in treatment outcomes. The efficacy of the commonly used chemotherapeutic agent, cisplatin, is diminished in patients with poor prognoses.

**Methods:**

Various bioinformatics databases were utilized to examine Carboxylesterase 1 (CES1) gene expression, clinicopathologic features, patient survival analysis, and gene function. An organoid model of HNSCC was established, along with the induction of drug-resistant HNSCC in the organoid model. CES1 expression was assessed using qRT-PCR and Western Blot, and differential markers were identified through transcriptome sequencing. Knockdown and overexpression models of CES1 were created in SCC-9 and patient-derived organoid (PDO) cells using shRNA and lentivirus to investigate the tumor biology and cisplatin resistance associated with CES1.

**Results:**

Research in bioinformatics has uncovered a strong correlation between the expression level of CES1 and the prognosis of HNSCC. The data suggests a significant link between CES1 expression and tobacco smoking. RNA-sequencing revealed a notable increase in CES1 expression in HNSCC-PDO^cis-R^ cells compared to the parental PDO cells. Subsequently, we performed *in vitro* studies by HNSCC-PDO and SCC-9 and found that CES1-overexpressing cells exhibited reduced sensitivity to cisplatin and stronger tumor malignant biological behavior compared with CES1-knockdown cells.

**Conclusion:**

The observed association between CES1 expression and tobacco smoking implies a potential influence of smoking on the efficacy of cisplatin-based chemotherapy in HNSCC through the regulation of CES1 expression.

## Introduction

Head and neck squamous cell carcinoma (HNSCC) is a malignant tumor that originates from the mucosal epithelium in the pharynx, larynx, oral cavity [1], and other areas. It ranks as the sixth most common malignancy globally [2], with approximately 700,000 new cases and 400,000 deaths reported annually [3]. The development of HNSCC is closely linked to risk factors such as tobacco smoking, alcohol consumption, environmental pollution, and viral infections like Epstein-Barr Virus (EBV) and human papillomavirus (HPV) [4,5]. Treatment options for HNSCC typically involve surgery, radiotherapy (RT), and chemotherapy (CT). Recent advancements in therapy have introduced monoclonal antibodies like cetuximab targeting the epidermal growth factor receptor (EGFR) and checkpoint inhibitors, such as those targeting PD1 or PDL1 [6,7] as additional strategies. Implementing a definitive multidisciplinary team (MDT) approach can act as a pivotal point for identifying improved strategies and enhancing clinical outcomes and patient quality of life in HNSCC. Despite these advancements, the prognosis for HNSCC patients, particularly those in advanced stages, remains poor. The overall 5-year survival rate hovers around 60% [8], dropping to 40% for patients with locally advanced disease [4]. Many factors contribute to the unfavorable outcomes of HNSCC, including age, gender, clinical stage, alcohol and tobacco use [9,10], tumor location, comorbidities, HPV infection, genetic influences [11], and treatment response.

In recent years, there has been increasing focus on the relationship between metabolic status and the effectiveness of cancer treatment [12,13]. Metabolic reprogramming is known to play a significant role in the initiation and progression of malignant tumors. The metabolism of glucose, amino acids, lipids, and other pathways plays a crucial role in therapeutic outcomes and disease prognosis. For instance, dysregulated lipid metabolism has been linked to the development and advancement of malignant tumors, as rapidly dividing cancer cells have a high demand for nutrients such as fatty acids and cholesterol [14]. Studies have shown that lipid droplets within tumor cells can stimulate tumor growth through various signaling pathways [15], including alterations in lipid metabolism that increase tumor susceptibility and bioactivity [16]. Lipid products also impact response to chemotherapy, influencing treatment outcomes [17,18].

Carboxylesterase 1 (CES1), a key enzyme in lipid metabolism, is a vital member of the serine hydrolase superfamily primarily located in the endoplasmic reticulum of tissue cells. CES1 exhibits a broad range of substrate specificities, capable of hydrolyzing various substrates containing esters, thioesters, carbamates, and amide bonds [19–21] as well as triacylglycerol [22]. Additionally, CES1 has been found to possess hydrolase activity against endogenous cannabinoids and prostaglandin glycerides [23]. Dysregulation of CES1 has been associated with the development of diseases such as diabetes, obesity, and cancer [24,25], with abnormal expression of CES1 observed in various cancers including liver [26], lung [27], colorectal [28], and prostate [25] cancer.

Despite limited research on the role of CES1 in malignant tumors, this study utilizes bioinformatics and cell molecular biology experiments to investigate the significance of CES1 in HNSCC, and its potential as a prognostic marker. By delving into the prognostic mechanisms of this aggressive tumor, this research aims to provide deeper insights into HNSCC progression and prognosis.

## Materials and Methods

### Data acquisition and processing

This study involved extracting CES1-related RNA-seq expression data and clinical information from The Cancer Genome Atlas (TCGA) website for HNSCC [29]. A total of 502 HNSCC patient samples and 44 adjacent normal tissue specimens were selected based on criteria such as age, sex, race, smoking history, clinical stage, and chemoradiotherapy. Gene expression data from RNA-seq were further analyzed, along with clinical features like TNM stage, clinical stage, histological grade, age, sex, smoking history, drinking history, and radiotherapy history. The mRNA expression data were presented as $\bar{\text{x}}$ ± SD. Pearson correlation analysis was conducted to assess the relationship between CES1 expression levels and immune checkpoint gene expression. Additionally, the expression of CES1 in HNSCC was explored through Gene Expression Profiling in the Gene Expression Omnibus (GEO) database [30].

### Database application

Timer-based [31] analysis was used to evaluate the differential expression of CES1 in different tumor types. The association between CES1 expression in HNSCC and clinical outcomes was assessed using the Kaplan-Meier Plotter [32] website. Survival analysis curves were generated to analyze patient prognosis based on clinical stages, TNM stage, malignancy degree, and CES1 expression level (high or low) in the TCGA dataset. The TNM staging system utilized in this study was based on the 8th edition of the American Joint Committee on Cancer (AJCC, 2017). Hazard ratios (HRs) and *p*-values were calculated with a 95% confidence interval.

Differential expression genes associated with CES1 were analyzed from the TCGA HNSCC dataset using the LinkFinder module of the Linkedomics website [33]. Pearson correlation coefficients were utilized to determine correlations, which were then visually represented in volcano maps and heat maps. Gene-related signaling pathways were enriched through gene enrichment analysis (GSEA). Additionally, the Gene Expression Profiling Interactive Analysis (GEPIA) [34] module was employed to predict the top 50 genes positively and negatively associated with CES1, allowing for an analysis of its functional interaction.

In this study, a total of 50 CES1-interacting proteins were identified through STRING [35] protein cross-network analysis. Co-expression and interaction genes of CES1 were examined using Venn diagrams, and the potential function of CES1 was investigated through enrichment analysis.

### Cell culture

Human HNSCC cell SCC-9 was maintained long-term in the Guangxi Key Laboratory for early prevention and treatment of high-incidence tumors. The cell culture medium consisted of 89% DMEM/F12 medium, 10% serum, and 1% penicillin, and the cells were cultured in a 37°C 5% CO_2_ cell incubator.

Human HNSCC patient-derived organoids (HNSCC-PDO) were generated using discarded surgical samples from Guangxi Medical University Cancer Hospital. The process involved grinding, lysing, cleaning, and centrifuging, the single cells, which were then embedded in Matrigel (Corning, 356231) and overlaid with pre-warmed DMEM/F12 advanced media containing specific growth factors and inhibitors. The HNSCC-PDOs were cultured in a 37°C 5% CO_2_ cell incubator. The same HNSCC PDO was later induced into cisplatin-resistant PDO (PDO^cis-R^) after prolonged cisplatin treatment. The study was approved by the Guangxi Medical University Medical Ethics Committee (20220110), and informed consent was obtained from the patients or their relatives.

### RNA-seq

PDO and PDO^cis-R^ samples were collected, and total RNA was extracted. RNA quality control was conducted using Agilent2100 (RNA ≥ 200 ng, 28S/18S ≥ 1.0, RIN ≥ 7.0). cDNA libraries were constructed for RNA-seq analysis using the Omicsmart platform (China). Clean reads were filtered and aligned to the reference genome (GRCh38), and mRNA expression levels were normalized using the FPKM method. Differential expression genes (DEGs) were identified using the Omicsmart online tool, with screening criteria set at |log_2_-fold change| > 0.5 and *p*–value < 0.05. Visualization of results was performed using R software (v4.0.3).

### Overexpression or knockdown of CES1

Lentiviral and shRNA constructs were obtained from Sangon Biotech (Shanghai, China). A lentiviral vector overexpression CES1 with puromycin resistance was constructed and packaged into recombinant lentivirus. SCC-9 cells and HNSCC-PDO were stably transfected after puromycin screening for two weeks following infection at a multiplicity of infection (MOI) of 10 (SCC-9) and 40 (HNSCC-PDO) for 6 h. Cells infected with an empty lentivirus served as the negative control. Three shRNAs (Table 1) targeting CES1 were designed and transfected into SCC-9 cells using the Invitrogen™ Neon™ Transfection System with Lipofectamine 3000 reagent. Fresh medium was replaced 6 h post-transfection, and efficiency along with over-expression was assessed after 72 h.

**Table 1:** shRNA sequences

Number	Gene symbol	Sequence	
		Sense (5′-3′)	Antisense (5′-3′)
1	ShCES1-411	CCUGCUGACUUGACCAAGAAATT	UUUCUUGGUCAAGUCAGCAGGTT
2	ShCES1-1085	CGGAAUUAACAAGCAGGAGUUTT	AACUCCUGCUUGUUAAUUCCGTT
3	ShCES1-1718	CCAGACAGAACACAUAGAGCUTT	AGCUCUAUGUGUUCUGUCUGGTT

Subsequently, SCC-9 and PDO were divided into four subgroups for functional validation post-transfection: CES1 overexpression (CES1-OE), CES1-OE-negative control (CES1-OE-NC), shRNA of CES1 (shCES1), and shCES1-negative control (shCES1-NC).

### Cell proliferation and cisplatin toxicity assay

For the toxicological assay, cells from these subgroups were seeded in a 96-well plate and treated with varying concentrations of cisplatin. CCK-8 assay was performed after 2 days, and the inhibition rate was calculated based on OD values at 450 nm using a microplate reader. IC50 values were determined from the suppression curve.

In the proliferation assay, cells from each group (CES1-OE, CES1-OE-NC, shCES1, and shCES1-NC) were cultured in 96-well plates at a density of 1000 cells per well in quintuplicate. The CCK-8 solution was added on days 1, 2, 3, 4, and 5, followed by a 2h incubation period. The absorbance at 450 nm was measured using a microplate reader to plot growth curves based on the formula: actual OD value = Experimental group OD value/Control Group OD value. CellTiter-Glo 3D Cell Viability Assay (Promega, G968B) was utilized to assess the viability of PDO cells. The CellTiter-Glo3D reagent was diluted 1:1 in a medium and added to the samples. Data could be collected within 30 min post-reagent addition, offering a quicker alternative to Chemiluminescence viability assays. The data analysis method remained consistent with the previous approach.

### Cell migration

For the IBIDI cell scratch assay a plug-in was positioned at the center of a 12-well plate and seeded with 3.5 × 10^4^ cells per well in triplicate. After observing overnight cell growth in the chamber, the plug-in was removed once confluence reached 90%. Following two washes, the plug-in was placed in reduced serum medium (2% FBS + 98% DMEM-F12) and incubated at 37°C 5% CO_2_ Cell incubator. Cell migration was monitored at time points 0, 4, 8, 12, and 16 h using an inverted microscope, with images captured for analysis. The wound healing area was quantified using Image J software and the wound healing rate was calculated as wound healing rate = (0 h area–12 h area)/0 h Area × 100%.

### Cell invasion

The Corning Transwell chamber was utilized to assess the invasive potential of tumor cells. In this experiment, matrigel was combined with DMEM-F12 basal medium at a ratio of 1:30, and the resulting diluted matrigel was evenly spread into the Transwell chamber at 100 μL per well. The chamber was then incubated at 37°C 5% CO_2_ for 2 h until the matrigel solidified. Subsequently, 5 × 10^4^ cells in 200 μL of serum-free medium were introduced into the upper chamber, while DMEM-F12 complete medium was added to the lower chamber. After 48 h, the Transwell chambers were removed, washed, fixed with 4% tissue fixative for 15 min, and stained with 0.1% crystal violet for 10 min. Following drying, cells that had migrated through the chambers were observed and counted under an inverted microscope.

### Statistical analysis

The study compared CES1 gene expression levels among groups using a *t*-test, and compared gene expression levels between HNSCC tissues and adjacent normal tissues using a paired *t*-test. Various statistical tests including the Mann-Whitney *u*-test, Fisher Test, chi-square test, and logistic regression analysis, were employed to investigate the relationship between clinicopathological features and CES1 expression. Pearson’s test was utilized to analyze genes highly correlated with CES1, the correlation of immune cells, and differential markers of transcriptome sequencing. GraphPad Prism 7 was used for statistical analysis and graphing in this study, presenting metrological data as mean ± standard deviation ($\bar{\text{x}}$±s) and comparing two metrological data sets using two independent-sample *t*-tests. Results were considered to be significant when *p* < 0.05.

## Results

### Abnormally bipolar level of CES1 expression in HNSCC

**Figure 1:**
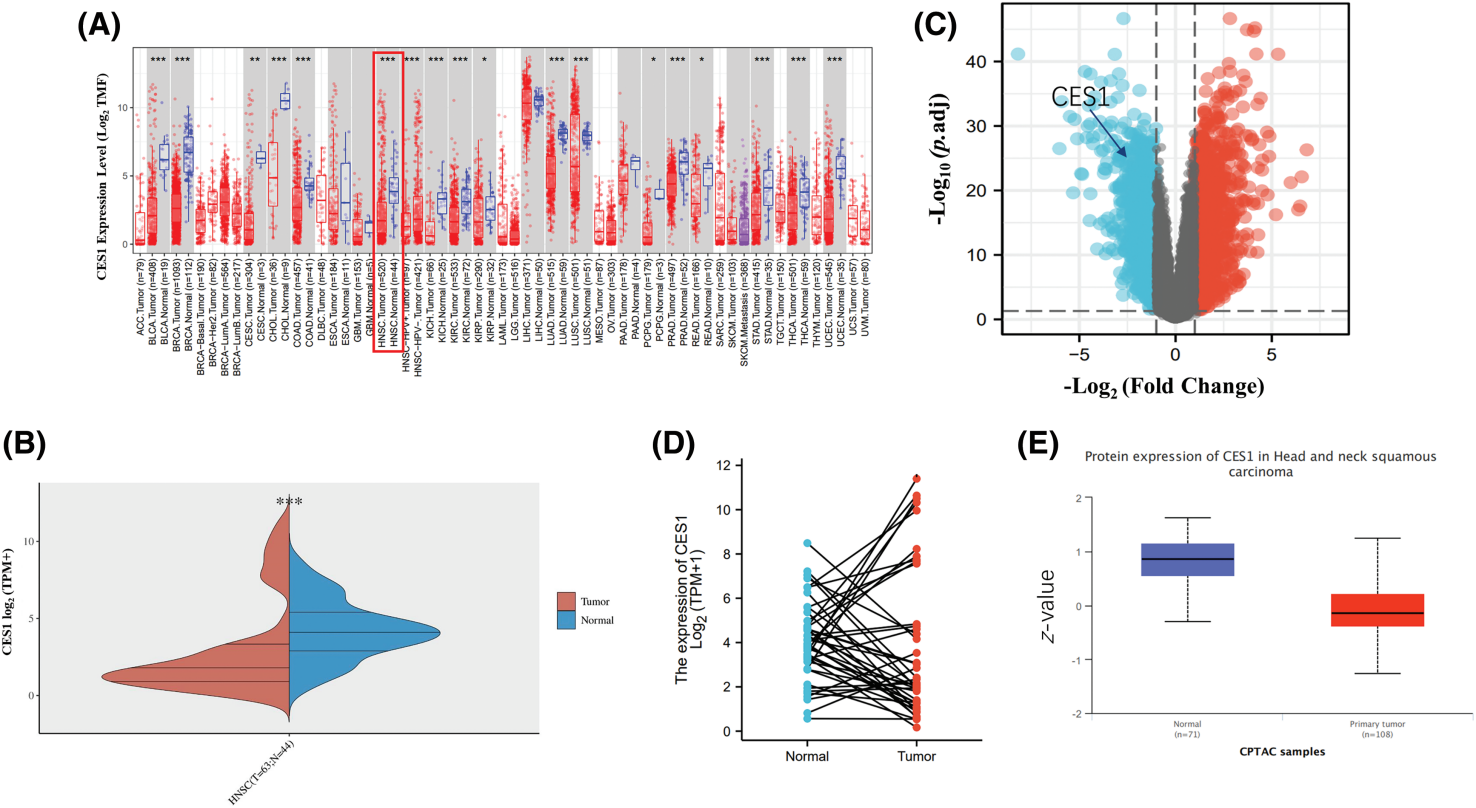
Abnormally bipolar level of CES1 expression in HNSCC. (A and B) The expression level of CES1 in pan-cancer derived from TCGA data. (C) Volcano plots of the DEGs in GEO. (D) Differential levels of CES1 mRNA expression in HNSCC samples and matched normal para-cancer samples. (E) The expression level of CES1 protein according to CPTAC. All data are presented as mean ± SEM, *p < 0.05, **p < 0.01, ***p < 0.001 ,****p < 0.0001.

The TIMER website was utilized to analyze CES1 mRNA expression. Results from Figs. 1A and 1B demonstrated a significant reduction in CES1 mRNA expression across various human cancers, including bladder, cholangiocarcinoma, renal cell carcinoma, and endometrial cancer. Interestingly, CES1 mRNA expression exhibited a bimodal distribution in certain cancers like HNSCC, lung squamous-cell carcinoma, hepatocellular carcinoma, and thyroid cancer. Subsequently, differential gene expression analysis in HNSCC was conducted using dataset GSE30784 from the GEO database, comparing 167 HNSCC patients with 62 normal controls. Fig. 1C illustrated the identification of 1898 up-regulated genes and 1732 down-regulated genes (*p* < 0.05). The analysis revealed an overall low expression trend of CES1 in the samples identified (|log_2_-fold change| > 1, *p* < 0.05). Paired data analysis in Fig. 1D showed a polarized expression of CES1 mRNA levels in HNSCC tissues (N = 43) compared to para-cancer normal tissues (N = 43). Furthermore, the study utilized the CPTAC section of the UALCAN online database to assess CES1 protein expression. Fig. 1E indicates a significantly lower expression of CES1 protein in HNSCC compared to normal tissues. Despite the general low expression of CES1 in HNSCC patients, some individuals exhibited high CES1 expression (Figs. 1A–1C). Consequently, the search team has developed a keen interest in investigating the role and mechanism of CES1 in HNSCC patients.

### Correlation of clinical and pathological features with CES1

**Figure 2:**
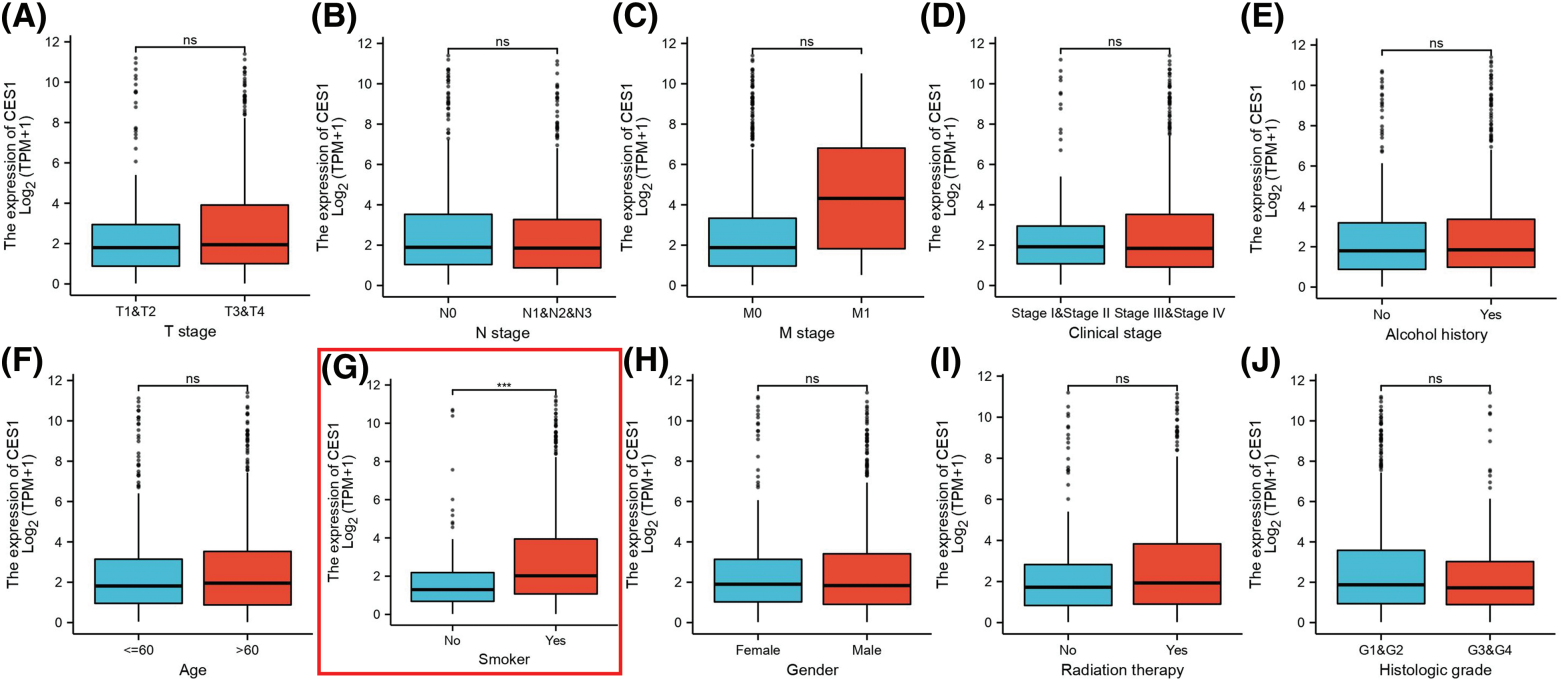
Clinical pathological characteristics correlated with CES1 mRNA expression levels using the TCGA dataset. (A) Age (B) alcohol history (C) clinical stage (D) gender (E) histologic grade (F) radiation therapy (G) smoker (H) T stage (I) N stage (J) M stage. All data are presented as mean ± SEM, *p < 0.05, **p < 0.01, ***p < 0.001, ****p < 0.0001.

The study investigated the association between CES1 expression and clinicopathological characteristics of HNSC tissues using the Mann-Whitney U test. Analysis of Figs. 2A–2J revealed a significant correlation between high CES1 expression and smoking history (*p* < 0.001), while no significant relationships were found with other clinical parameters like T stage, N stage, M stage, clinical stage, age, sex, or alcohol history. Additionally, the univariate Logit model in Table 2 demonstrated a strong association between CES1 expression and smoking history [OR = 2.285 (1.479–3.578). However, T stage [OR = 1.089 (0.753–1.577)], N stage [OR = 0.951 (0.665–1.360)], M stage [OR = 1.450 (0.238–11.083)], and clinical stage [OR = 0.860 (0.564–1.308)] did not show significant correlations.

**Table 2:** CES1 correlated with clinicopathological features of HNSCC

Characteristics	Total (N)	Odds Ratio (OR)	*p*
T stage (T3&4 *vs*. T1&2)	487	1.089 (0.753–1.577)	0.650
N stage (N1&2&3 *vs*. N0)	480	0.951 (0.665–1.360)	0.783
M stage (M1 *vs*. M0)	477	1.450 (0.238–11.083)	0.685
Clinical stage (Stage3&4 *vs*. Stage1&2)	488	0.860 (0.564–1.308)	0.480
Radiation therapy (Yes *vs*. No)	441	1.210 (0.818–1.794)	0.340
Gender (Male *vs*. Female)	502	0.922 (0.620–1.369)	0.687
Age (>60 *vs*. ≤60)	501	1.261 (0.888–1.793)	0.195
Histologic grade (G3&4 *vs*. G1&2)	483	0.891 (0.589–1.344)	0.581
Smoker (Yes *vs*. No)	492	2.285 (1.479–3.578)	<0.001
Alcohol history (Yes *vs*. No)	491	1.019 (0.698–1.490)	0.920

### Poor prognosis of HNSCC patients is related to the high expression of CES1

**Figure 3:**
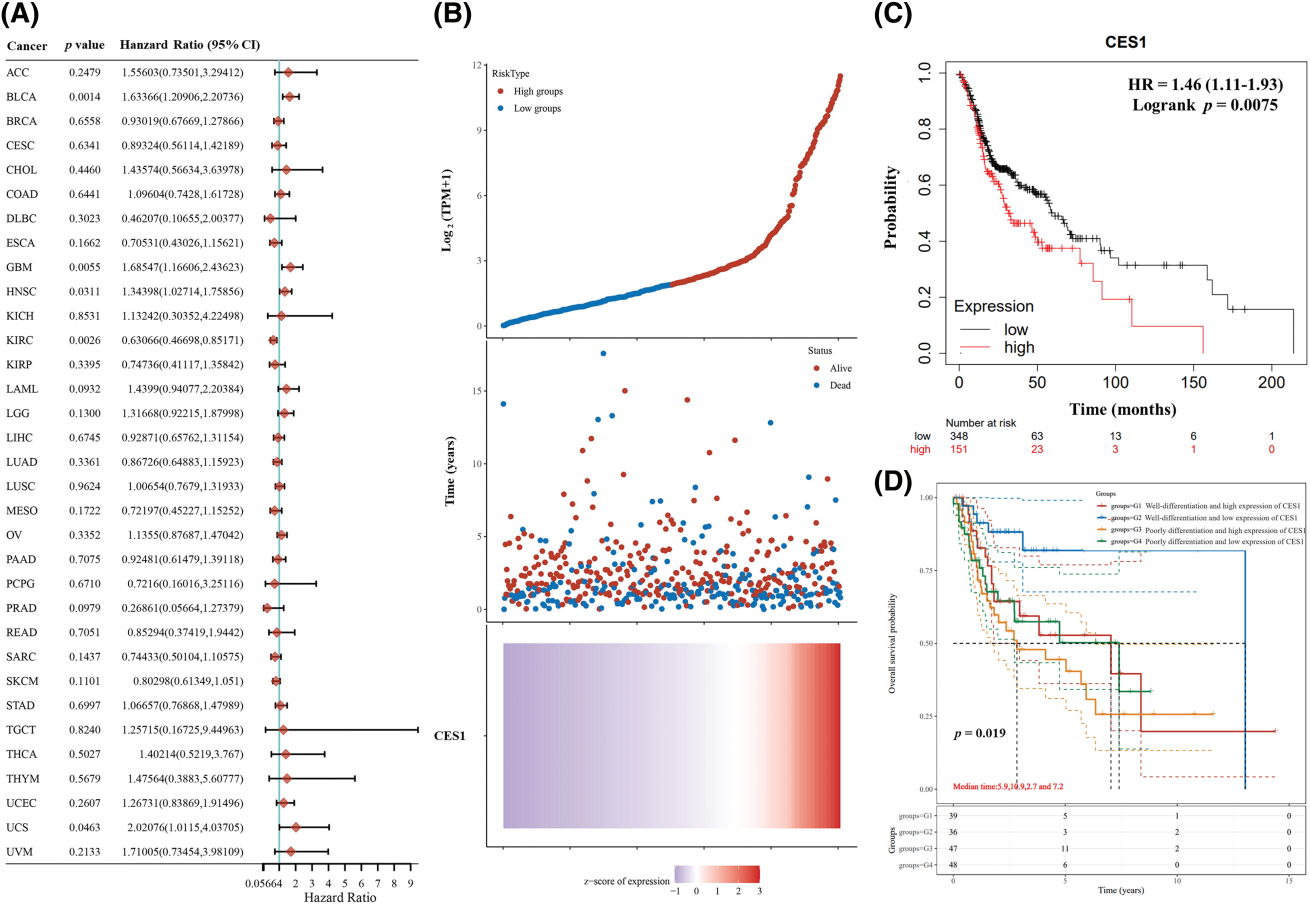
Poor prognosis of patients with HNSCC is related to the high expression of CES1. (A) Forest plot of survival analysis curves for CES1 in pan-cancer in the TCGA patient database. (B, C) Survival analyses of the relationship between HNSCC patients with high and low CES1 expression. (D) Tumor differentiation and CES1 expression in patients with survival analysis from TCGA database. All data are presented as mean ± SEM, *p < 0.05, **p < 0.01, ***p < 0.001, ****p < 0.0001.

Kaplan-Meier charts and curves were utilized to analyze the correlation between CES1 mRNA expression and overall survival (OS) in patients with HNSCC. The analysis of TCGA tumor patient data revealed that high CES1 expression was associated with poor prognosis in various types of tumors including adrenocortical carcinoma, bladder cancer, HNSCC, lung squamous cell carcinoma, and hepatocellular carcinoma (Fig. 3A). Particularly in HNSCC (Fig. 3B), CES1 was found to have low overall expression, with abnormally high levels indicating a significant risk factor for poor prognosis (Fig. 3C), with a hazard ratio (HR) of 1.46 (1.11–1.93) and a *p* = 0.0075. Furthermore, grouping TCGA-HNSCC patient data based on clinical stage and CES1 expression levels into four categories (G1, G2, G3, G4) revealed varying survival times of 5.9 years, 10.9 years, 2.7 years, and 7.2 years, respectively (Table 3), for 50% survival rate. CES1’s adverse prognostic impact on HNSCC patients was found to be more significant than clinical staging alone (Fig. 3D). Overall, high CES1 expression may serve as a prognostic indicator for poor outcomes in HNSCC patients, warranting further investigation into the underlying mechanisms.

**Table 3:** Multi-group survival analysis characteristics

Group	Characteristics	Total (N)
G1	Well-differentiation and high expression of CES1	39
G2	Well-differentiation and low expression of CES1	36
G3	Poor-differentiation and high expression of CES1	47
G4	Poor-differentiation and low expression of CES1	48

### Prediction of CES1 function in HNSCC

**Figure 4:**
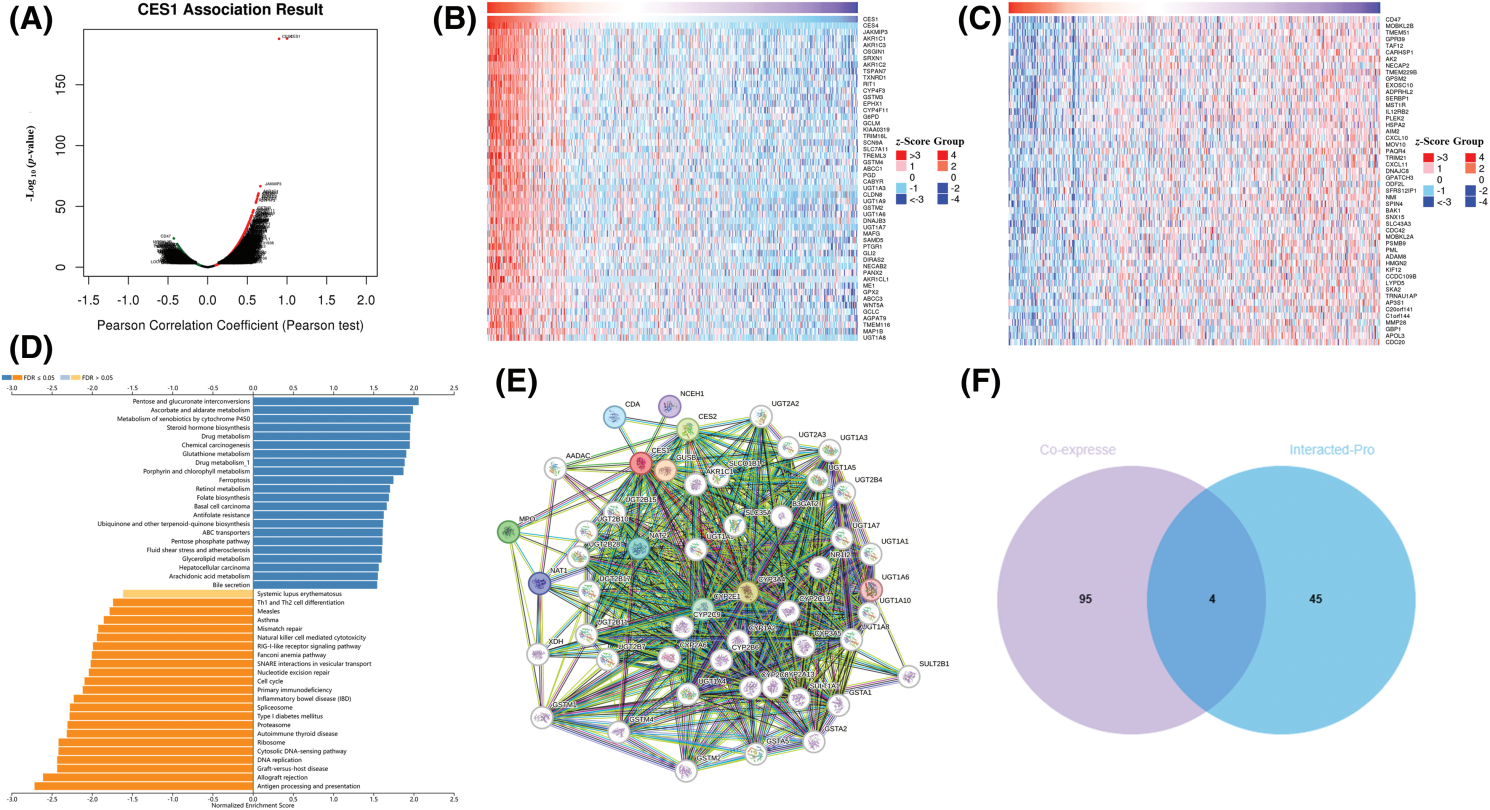
Prediction of CES1 function in HNSCC based on linkedomic online site. (A) CES1-related genes were screened from Linkemodic database for enrichment analysis. (B) Genes positively correlating with CES1. (C) Genes negatively correlating with CES1. (D) Result graph of CES1 enrichment analysis. (E) A network visualization for CES1-binding proteins was created using the STRING database. (F) Venn diagram cross-referenced CES1-related genes and interacting proteins.

The study investigated co-expressed genes with CES1 in TCGA-HNSCC using the LinkFinder section of the Linkedomics online database to gain insights into the biological function of CES1. In Fig. 4A, a total of 7951 genes were found to have a positive correlation with CES1, while 3485 genes showed a negative correlation. Figs. 4B and 4C displayed heatmaps illustrating the top 50 genes positively and negatively linked to CES1 expression levels, respectively. Notably, among the top 50 genes positively associated with CES1, forty-one were identified as poor prognostic markers (HRs > 1) in HNSCC patients. Conversely, among the top 50 genes negatively correlated with CES1, thirty-four were identified as protective factors (HRs < 1) in HNSCC. Recent studies on oral squamous-cell carcinoma have highlighted the effectiveness of ND-MN Molecular Cluster screening by oral cancer imaging [36] and targeted therapy using nanomaterials combined with antibodies in cancer patients [37,38]. Subsequently, KEGG enrichment analysis was conducted to explore CES1-related signaling pathways. In Fig. 4D, pathways significantly positively correlated with CES1 included pentose-glucuronic acid conversion, ascorbic acid, and aldehydes metabolism, cytochrome P450 signaling pathway, porphyrin and chlorophyll metabolism, chemical carcinogenesis, steroid hormone biosynthesis, glutathione metabolism, drug metabolism, retinol metabolism, ATP binding cassette transporter family, among others. Conversely, pathways significantly negatively correlated with CES1 included allograft rejection, antigen processing presentation, DNA replication, graft-versus-host disease, cell cycle, cytosolic DNA sensing pathway, and proteasome synthesis.

To delve deeper into the role of the CES1 gene in tumorigenesis and development, we utilized the STRING online platform for protein interaction network analysis. Figs. 4E and 4F displayed a network involving 50 proteins that interact with CES1. Additionally, through a Venn diagram analysis, the study identified key genes such as ABCC1, CYP4F11, EPHX1, and UGT1A8 by intersecting the top 50 CES1 co-expressed genes with the top 50 interacting proteins.

**Figure 5:**
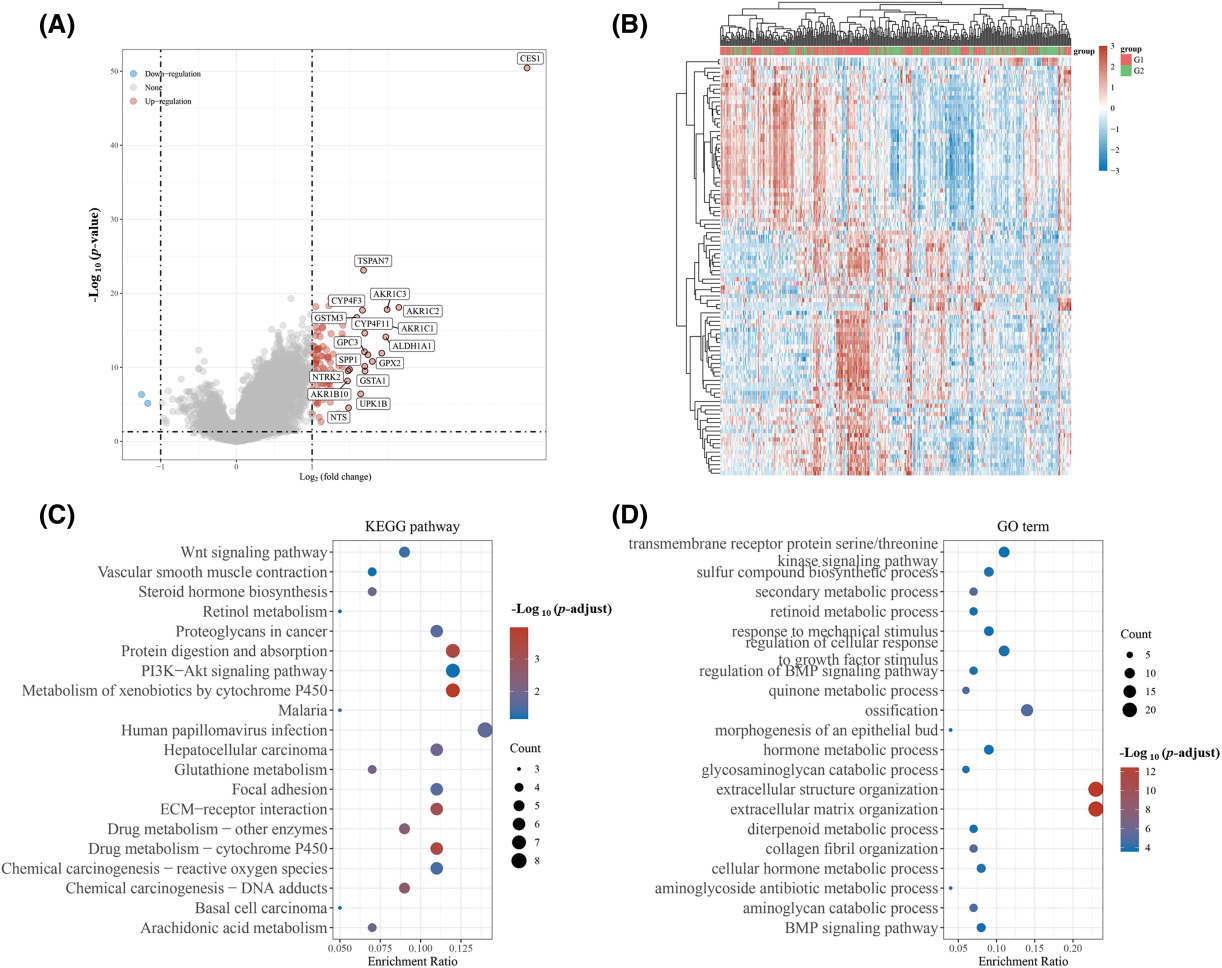
Differential genes were screened and CES1 functions were predicted based on the expression level of CES1 in HNSCC patients of TCGA. (A, B) Differential genes between patients with low CES1 expression and those with high CES1 expression in the TCGA database. (C) KEGG enrichment analysis of signaling pathway. (D) GO enrichment analysis of signaling pathway.

In this study, patients with HNSCC in the TCGA database were categorized based on CES1 expression levels into two groups: G1 (smokers with high CES1 expression) and G2 (smokers with low CES1 expression). Differential gene expression was then observed between these two groups to investigate underlying mechanisms, using KEGG and GO enrichment analyses. The volcano plot in Figs. 5A and 5B revealed ninety DEGs when comparing patients with high and low CES1 expression, with 88 upregulated genes and 2 down-regulated genes. Additionally, KEGG enrichment analysis (Figs. 5C and 5D) showed enrichment of differentially expressed genes in pathways such as Wnt signaling, retinol metabolism, platinum resistance, cytochrome P450 pathway, glutathione metabolism, drug metabolism, reactive oxygen species chemical carcinogenicity, arachidonic acid metabolism, among others. GO enrichment analysis revealed enrichment in pathways Like sulfur biosynthesis, retinoic acid metabolism, quinine metabolism, hormone metabolism, and BMP signaling pathway. Notably, platinum metabolism emerged as a significant pathway, potentially influencing drug metabolism, glutathione metabolism, and chemoresistance. Genes associated with chemoresistance, such as the ABC drug efflux family, Cytochrome CYP450 family, and NAT protein that interacts with CES1, were identified.

### CES1 was significantly up-regulated in a cisplatin-resistant HNSCC PDO

**Figure 6:**
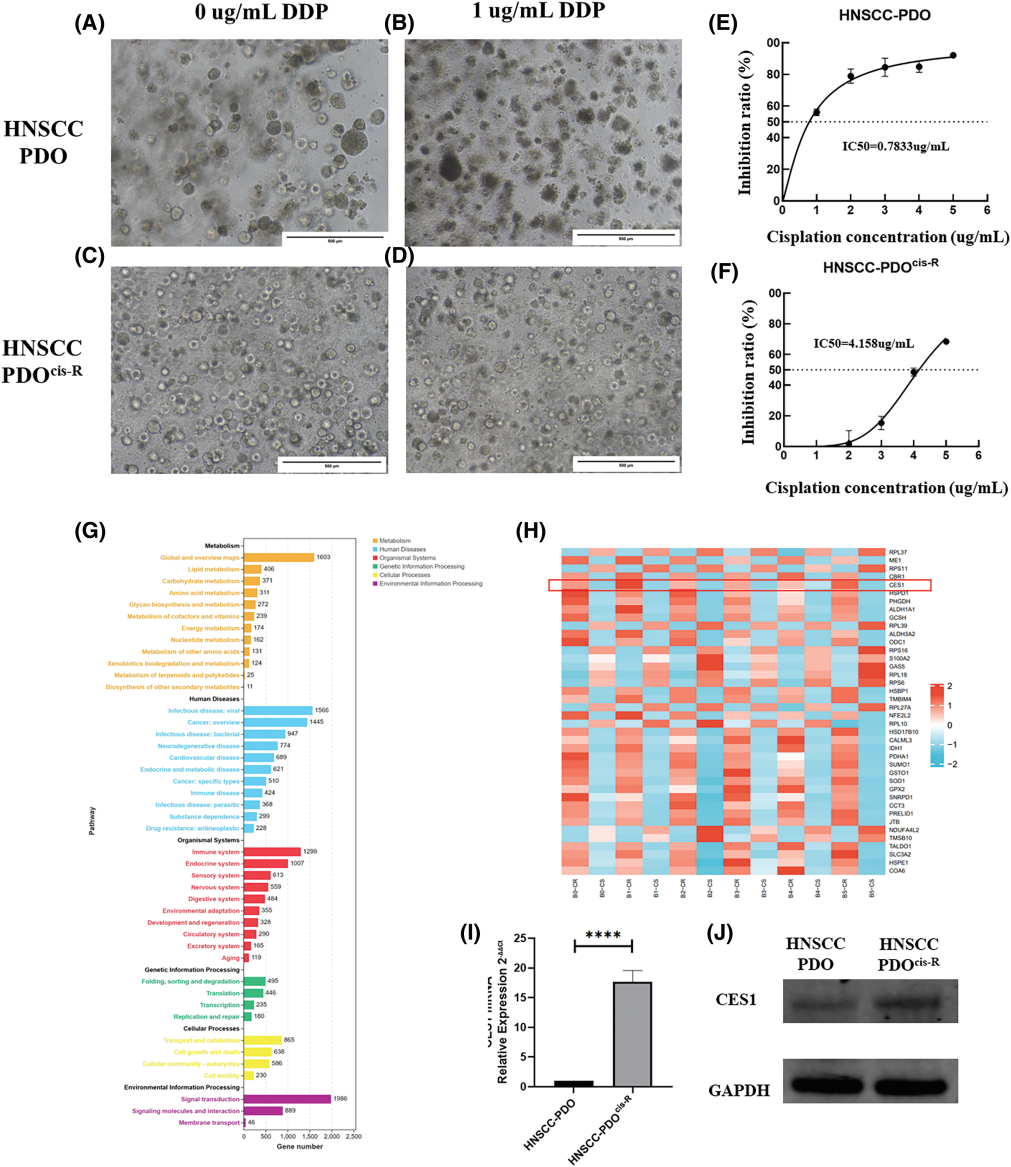
CES1 was significantly up-regulated in cisplatin-resistant PDO of HNSCC compared to the parental control. (A–D) The basal culture of HNSCC-PDO and HNSCC-PDO^cis-R^ and the culture condition after cisplatin treatment; (E, F) Detection of Cisplatin IC50 in HNSCC-PDO and HNSCC-PDO^cis-R^. (G, H) By comparing transcriptome sequencing results of PDO and PDO^cis-R^, the cisplatin resistance-related genes were screened and their functions were analyzed; (I) qRT-PCR showed that the expression of CES1 in the HNSCC-PDO^cis-R^ was significantly higher than that in the parental PDO. (J) The significantly higher level of CES1 expression in HNSCC-PDO^cis-R^ compared to parental HNSCC-PDO detected by WB detection. All data are presented as mean ± SEM, *p < 0.05, **p < 0.01, ***p < 0.001, ****p < 0.0001.

HNSCC PDO and HNSCC cisplatin-resistant PDO (HNSCC-PDO^cis-R^) were established and prepared for subsequent analyses. The successful construction of the PDOcis-R was verified by detecting the growth status and the IC50 value of cisplatin administration. Culturing HNSCC PDO (parental control) and HNSCC-PDO^cis-R^ for 48 h with a medium containing 0 and 1 μg/mL cisplatin revealed that the HNSCC-PDO exhibited more intense boundary cracking and cell death compared to the PDO^cis-R^, as depicted in Figs. 6A–6D. The IC50 value of cisplatin for the PDOcis-R (4.158 μg/mL) was notably higher than that for the parental PDO (0.783 μg/mL), with the former being 5.31 times greater than the latter (Figs. 6E and 6F). Potential chemoresistance-related genes were identified through transcriptome sequencing comparing PDO with PDO^cis-R^, followed by functional enrichment analysis. The results, illustrated in Figs. 6G and 6H, indicated that chemoresistance in HNSCC is primarily associated with lipid metabolism and carbohydrate metabolism at the metabolic level. In terms of human disease, differential gene functions were enriched in pathways related to virus infection, tumorigenesis, and drug resistance. Similarly, for organismal systems, differential gene functions were enriched in pathways associated with the immune system, endocrine system, and sensory system. Subsequent validation of CES1 expression at RNA and protein levels between HNSCC-PDO and HNSCC-PDO^cis-R^ using RT-PCR and WB revealed that the expression level of CES1 in HNSCC-PDO^cis-R^ was 17.697 times higher than in the PDO (Figs. 6I and 6J). WB results also showed significant up-regulation of CES1 in PDO^cis-R^ compared to its parental PDO. Additionally, analysis of the GSE102787 dataset in GEO highlighted CES1 as a significantly overexpressed gene linked to cisplatin resistance in HNSCC. This study suggests that CES1 could serve as a potential marker for cisplatin resistance in HNSCC.

### CES1 promoted proliferation, invasion, migration, and cisplatin resistance in HNSCC

**Figure 7:**
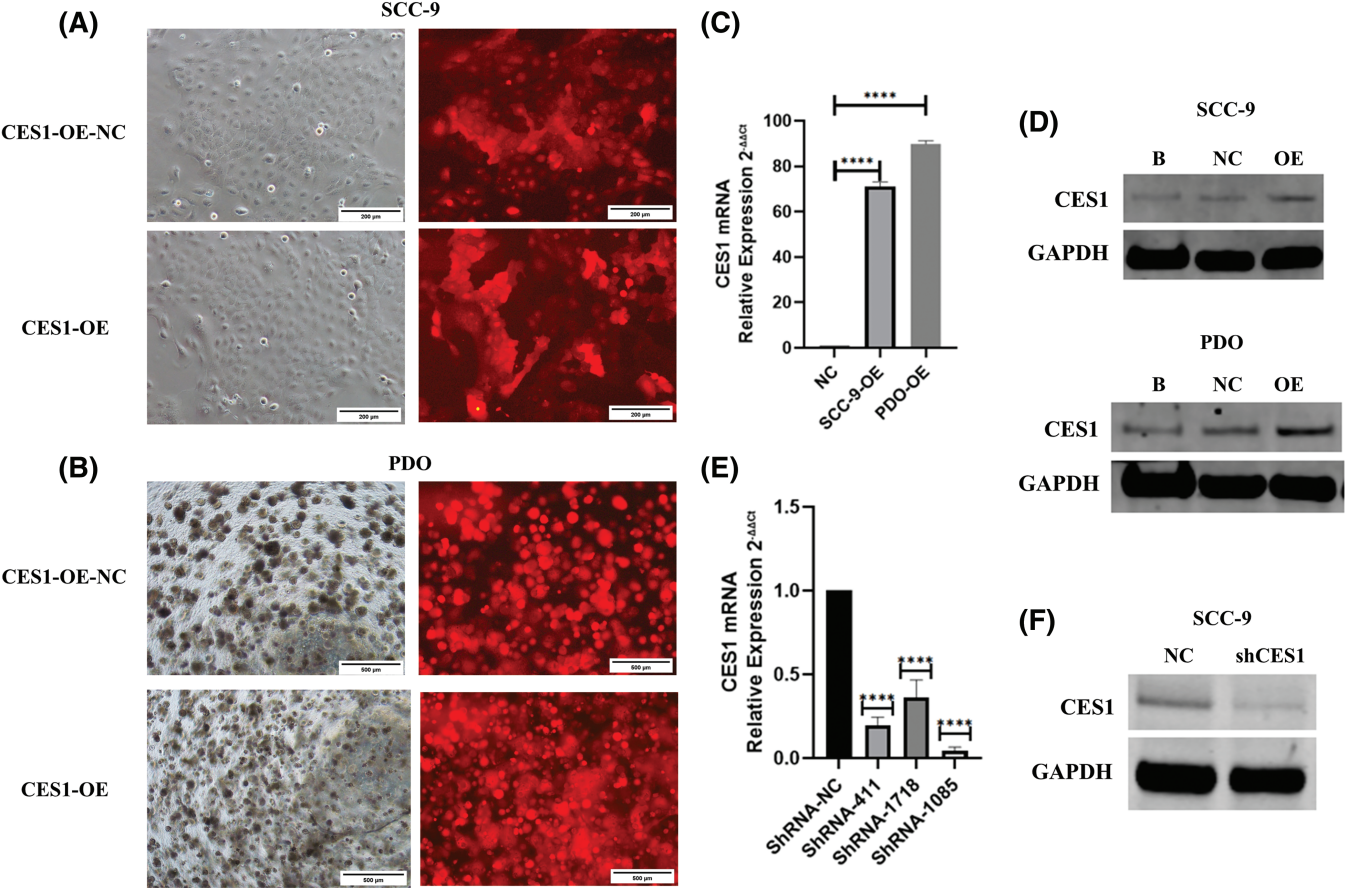
Construction of CES1 knockdown and overexpression model. (A and B) SCC-9 and HNSCC-PDO with over-expression and negative control were observed under the fluorescence microscope. (C) qRT-PCR was used to detect the expression of CES1 in SCC-9 and HNSCC-PDOs infected with lentivirus harboring CES1 or empty lentivirus. (D) Detection of CES1 expression by WB in SCC-9 and HNSCC-PDOs after infection with lentivirus harboring CES1 or empty lentivirus. (E) qRT-PCR was used to detect the efficiency of CES1 knockdown. (F) WB was used to detect the efficiency of CES1 knockdown. All data are presented as mean ± SEM, *p < 0.05, **p < 0.01, ***p < 0.001, ****p < 0.0001.

Based on the findings of this study, CES1 is implicated in the development and cisplatin resistance of HNSCC. Subsequent functional validation involved creating CES1-knockdown and overexpression models in SCC-9 and HNSCC PDO through shRNA transfection and lentiviral infection. Fluorescence microscopy confirmed successful lentiviral infection (Figs. 7A and 7B), with qRT-PCR and WB analysis showing significant upregulation of CES1 in the OE groups compared to controls (Figs. 7C and 7D). Moreover, CES1 expression was notably reduced in SCC-9 after shRNA transfection, with shRNA-1085 selected for further experiments due to its high knockdown efficiency (Figs. 7E and 7F).

**Figure 8:**
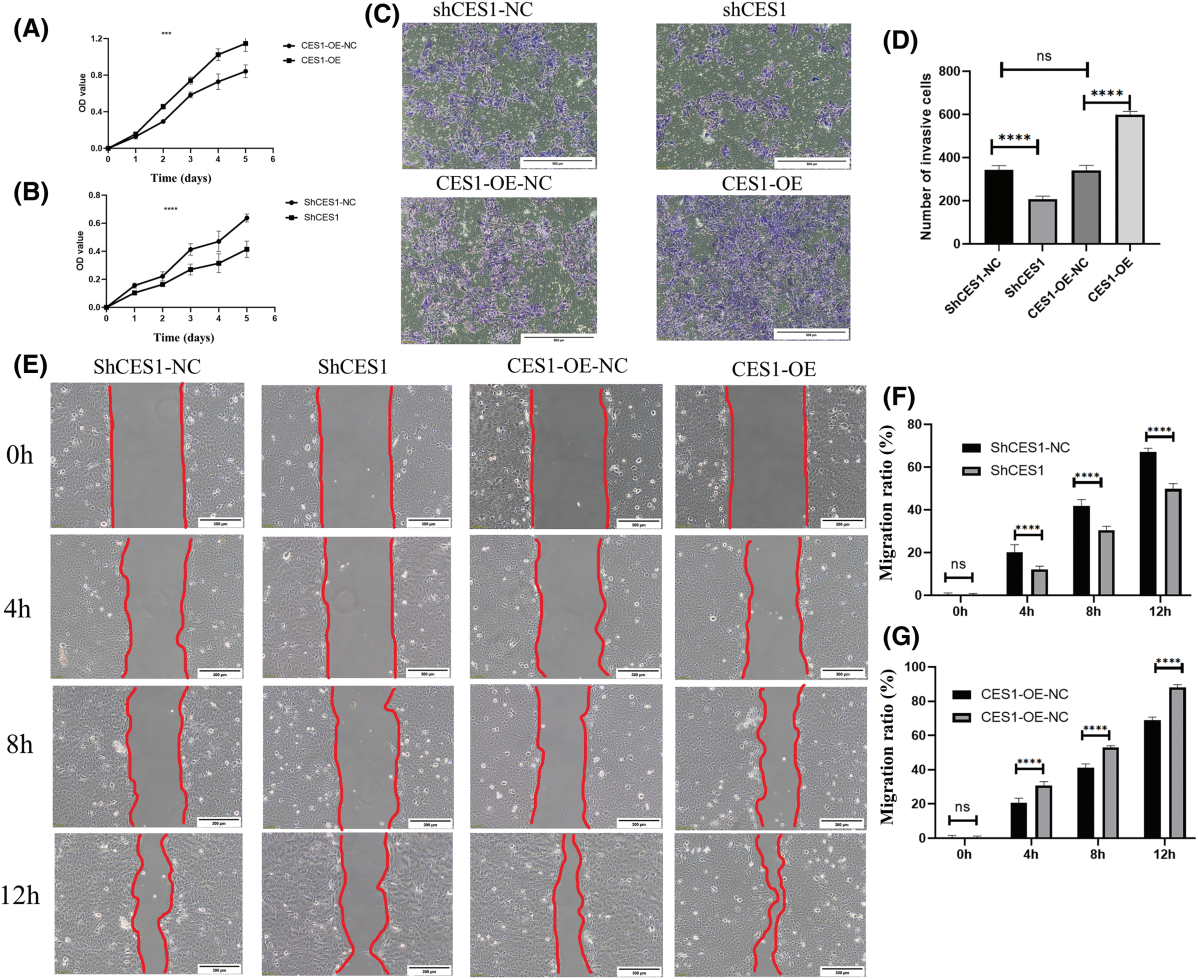
CES1 promotes SCC-9 cell proliferation, invasion, and migration. (A, B) SCC-9 proliferation 0–6 days after CES1 overexpression and CES1 knockdown using CCK8 kit. (C, D) Analyses of SCC-9 tumor invasion after CES1 overexpression and CES1 knockdown. (E–G) Scratch healing assay was used to detect SCC-9 tumor migration after CES1 overexpression and CES1 knockdown. All data are presented as mean ± SEM, *p < 0.05, **p < 0.01, ***p < 0.001, ****p < 0.0001.

Proliferation assay using the CCK-8 kit, cell scratch assay, and Transwell invasion assay were conducted to assess the impact of CES1 on the cellular behavior of different subgroups of SCC-9 cells, including CES1-OE-NC, CES1-OE, shCES1-NC, and shCES1. The results of the proliferation assay, depicted in Figs. 8A and 8B, demonstrated that CES1 overexpression significantly enhanced SCC-9 cell proliferation, while CES1 knockdown notably suppressed it, with both differences being statistically significant (*p* < 0.001). The outcomes of the transwell invasion experiments, presented in Figs. 8C and 8D, revealed that CES1 overexpression promoted SCC-9 invasion, whereas CES1 knockdown inhibited it, with statistical significance (*p* < 0.001). The findings from the scratch test (Figs. 8E–8G), showed complete healing of SCC-9 scratches within 12–16 h under low serum conditions. The migration extent between 0–12 h was measured and statistically analyzed, indicating that CES1 overexpression facilitated SCC-9 cell migration, while CES1 knockdown impeded it, with statistical significance (*p* < 0.001).

**Figure 9:**
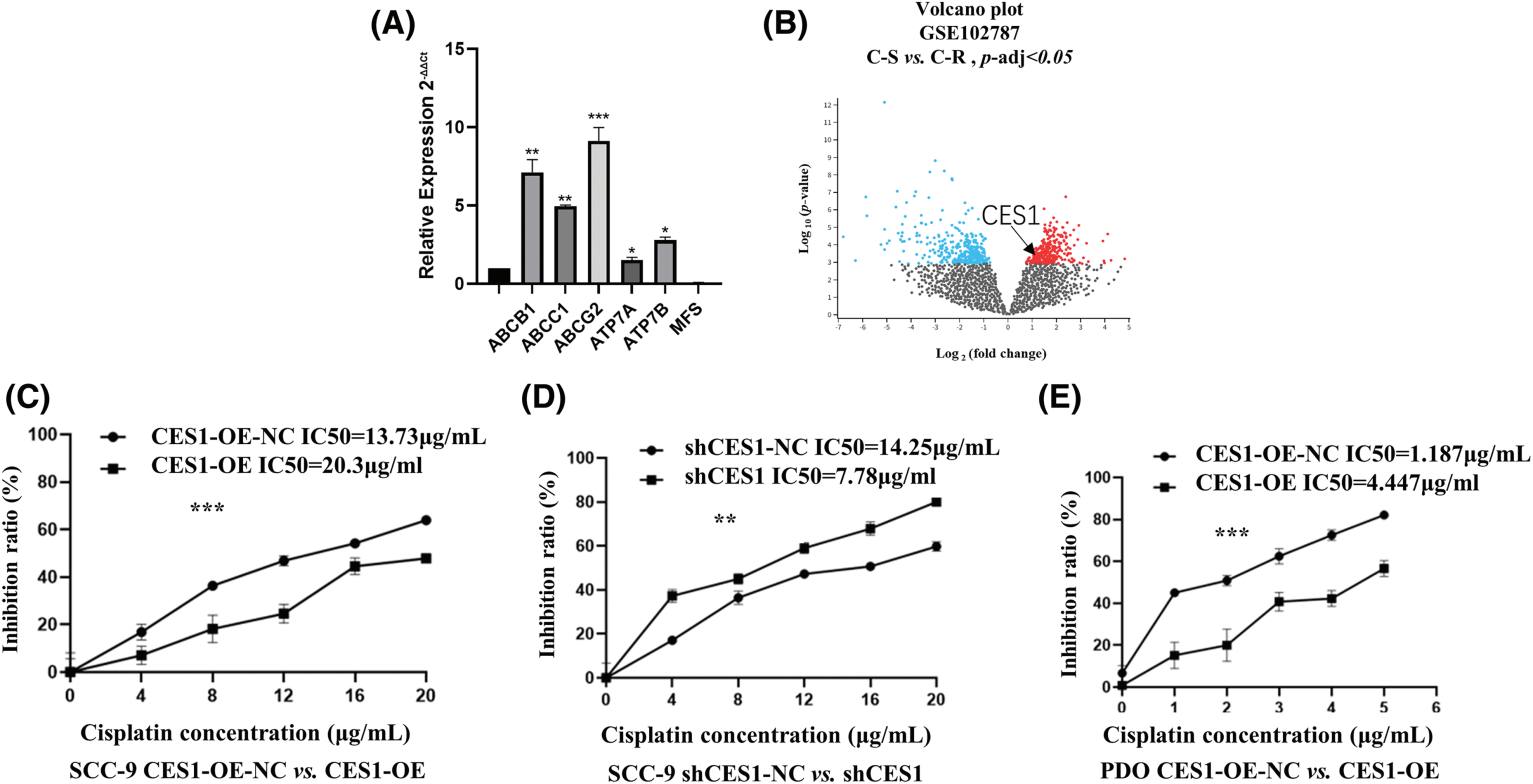
CES1 promotes cisplatin-resistance in HNSCC. (A) qRT-PCR was used to detect the up-regulation of chemoresistance-related genes regulated by CES1. (B) GSE102787 data in the GEO database shows that upregulated CES1 is associated with cisplatin resistance in HNSCC. (C–E) Cytotoxic assays demonstrated that overexpression of CES1 promoted cisplatin resistance in HNSCC, and that knockdown of CES1 promoted the sensitivity of HNSCC to cisplatin. All data are presented as mean ± SEM, *p < 0.05, **p < 0.01, ***p < 0.001, ****p < 0.0001.

qRT-PCR was utilized to identify genes associated with multidrug resistance in CES1-overexpressed SCC-9 cells, including ABCB1, ABCC1, ABCG2, ATP7A, ATP7B, and MFS. The expression levels of ABCB1, ABCC1, and ABCG2 were notably higher compared to the negative control, with values of 7.12 ± 0.81, 4.95 ± 0.07, and 9.13 ± 0.85, respectively. Conversely, ATP7A, ATP7B, and MFS exhibited lower expression values of 1.51 ± 0.18, 2.80 ± 0.18, and 0.045 ± 0.04, respectively (Fig. 9A). These results suggest that CES1 overexpression can enhance cisplatin resistance by modulating multidrug resistance mechanisms in HNSCC cells. Furthermore, analysis of the GSE102787 dataset from the GEO database revealed a significant upregulation of CES1 in cisplatin-resistant HNSCC, indicating its potential role as a cisplatin-resistant factor in HNSCC (Fig. 9B). Cisplatin cytotoxicity data for SCC-9 cells demonstrated a positive correlation between cisplatin concentration and inhibition rates across different treatment groups (CES1-OE-NC, CES1-OE, shCES1-NC, shCES1), as depicted in Figs. 9C and 9D. Specifically, Fig. 9D illustrated that CES1-OE cells exhibited substantial cisplatin resistance compared to CES1-OE-NC cells, while the shCES1-treated group displayed increased sensitivity to cisplatin compared to the shCES1-NC group. Additionally, analysis of cisplatin cytotoxicity in HNSCC-PDO samples revealed that CES1-OE PDO (Fig. 9E) exhibited greater cisplatin resistance (IC50 = 4.447 μg/mL) compared to CES1-OE-NC (IC50 = 1.187 μg/mL), underscoring the role of CES1 in promoting cisplatin resistance in HNSCC.

## Discussion

Most patients with HNSC are diagnosed in the middle or late stages. While cisplatin-based concurrent chemoradiotherapy is the standard first-line treatment for advanced HNSCC [39], the development of cisplatin resistance often leads to tumor recurrence, disease progression, and reduced overall survival. In this study, the bioinformatics analyses revealed that HNSCC patients with high CES1 expression had a poorer prognosis compared to those with low expression. Surprisingly, patients in late clinical stages with low CES1 expression had better outcomes than those in early stages with high CES1 expression, indicating CES1’s significance as an independent prognostic factor in HNSCC. Further investigation into the role of CES1 in HNSCC prognosis is warranted. The study found that high CES1 expression in HNSCC-PDO^cis-R^ was associated with decreased sensitivity to cisplatin in both cell lines and HNSCC-PDO. Knockdown of CES1 using shRNA increased drug cytotoxicity, suggesting a potential link between CES1 and cisplatin resistance in HNSCC.

The impact of CES1 on the sensitivity of HNSCC to cisplatin remains not completely understood, with a complex mechanism involving changes in cancer stem cell phenotype, epithelial-mesenchymal transition, drug efflux, autophagy, and metabolic reprogramming [39]. This study revealed that the multidrug resistance-related proteins ABCB1, ABCC1, ABCG2, ATP7A, and ATP7B were significantly up-regulated in the CES1-overexpressed SCC-9 cells. ABCB1, ABCC1, and ABCG2, are key members of the ATP-binding cassette (ABC) transporter family, which are crucial for multidrug resistance in cancer cells [40]. Additionally, ATP7A and ATP7B are copper transporters [41], which also play a role in drug transport, facilitating the export of cisplatin from cells, and thereby regulating its intracellular accumulation [42,43]. Previous studies have shown that the cytotoxicity of cisplatin is directly linked to its intracellular levels [41]. The findings of this study suggest that CES1 may enhance cisplatin efflux from HNSCC cells by up-regulating, ABCB1, ABCC1, ABCG2, ATP7A, and ATP7B, leading to reduced intracellular cisplatin levels and ultimately promoting cisplatin resistance in HNSCC. Lipid metabolism remodeling may also emerge as a critical mechanism in metabolic reprogramming that contributes to cisplatin resistance in HNSCC. Lipids, essential nutrients in the human body, undergo metabolic reprogramming during tumor progression. Tumor cells alter lipid metabolism to facilitate energy and lipid droplet accumulation, thereby mitigating the oxidative stress induced by chemotherapeutic agents and fostering resistance to chemotherapy [44]. CES1, as a key enzyme in lipid metabolism, plays a significant role in the onset and progression of various tumors. It has been documented to possess triacylglycerol hydrolase activity [45]. Additionally, CES1 has demonstrated hydrolase activity against endogenous cannabinoid 2-arachidonoylglycerol and its cyclooxygenase prostagl and in glycerides [23]. Prior research has indicated that lipid metabolic reprogramming is a key characteristic of tumor cell initiation and development [28]. Moreover, the normal upregulation of CES1, which activates the PPAR α-SCD signaling axis, is believed to enhance lipid metabolism and potentially contribute to cisplatin resistance in hepatocellular carcinomas [46]. Our findings suggest that CES1 may reduce the sensitivity of HNSCC to cisplatin by disrupting lipid metabolic pathways, providing initial evidence of CES1’s impact on the treatment outcome of HNSCC.

A significant positive correlation was observed in this study between high CES1 expression and smoking history in HNSCC patients using TCGA dataset analysis. It is well-known that tobacco smoking is closely associated with the occurrence, development, and prognosis of HNSCC [47,48]. Tobacco contains over 70 known carcinogens [49], which impact HNSCC risk and prognosis by interfering with metabolism-related enzymes such as those in the cytochrome P450 family, glutathione, glucuronosyltransferase, and aldehyde dehydrogenase [50]. The study revealed that genes regulated by CES1 were enriched in pathways such as cytochrome P450 signaling, glutathione metabolism, and glucuronic acid metabolism, indicating a combined effect of tobacco smoke and CES1. It was also noted that neuroactive substances in tobacco, like cannabis, 9-tetrahydrocannabinol (THC), and cannabidiol (CBD), could influence CES1 expression through an endogenous cannabinoid metabolic pathway [23,26]. These findings suggest that carcinogens in tobacco might increase the risk and worsen the prognosis of HNSCC by modulating CES1 expression.

In summary, CES1 may play a role in determining the prognosis of patients with HNSC through its impact on the effectiveness of cisplatin-based therapy. Factors like tobacco smoking could potentially worsen treatment outcomes in HNSCC patients by increasing the expression of CES1. However, this study lacks extensive experimental validation, and further research is needed to elucidate the specific mechanism of CES1.

## Data Availability

The data that support the findings of this study are available from the corresponding author, upon reasonable request.
